# Supplementary material for: Identifying repeat domains in large genomes
Source: Genome Biol. 2006 Jan 31;7(1):R7. doi: 10.1186/gb-2006-7-1-r7 (PMC1431705; doi:10.1186/gb-2006-7-1-r7)
Supplement: Additional File 1 — A zipped file of browsable HTML files with a complete list of the connected components in the repeat domain graph of human Repbase. [file gb-2006-7-1-r7-S1.gz › html/subgraphs/8789.pdf]

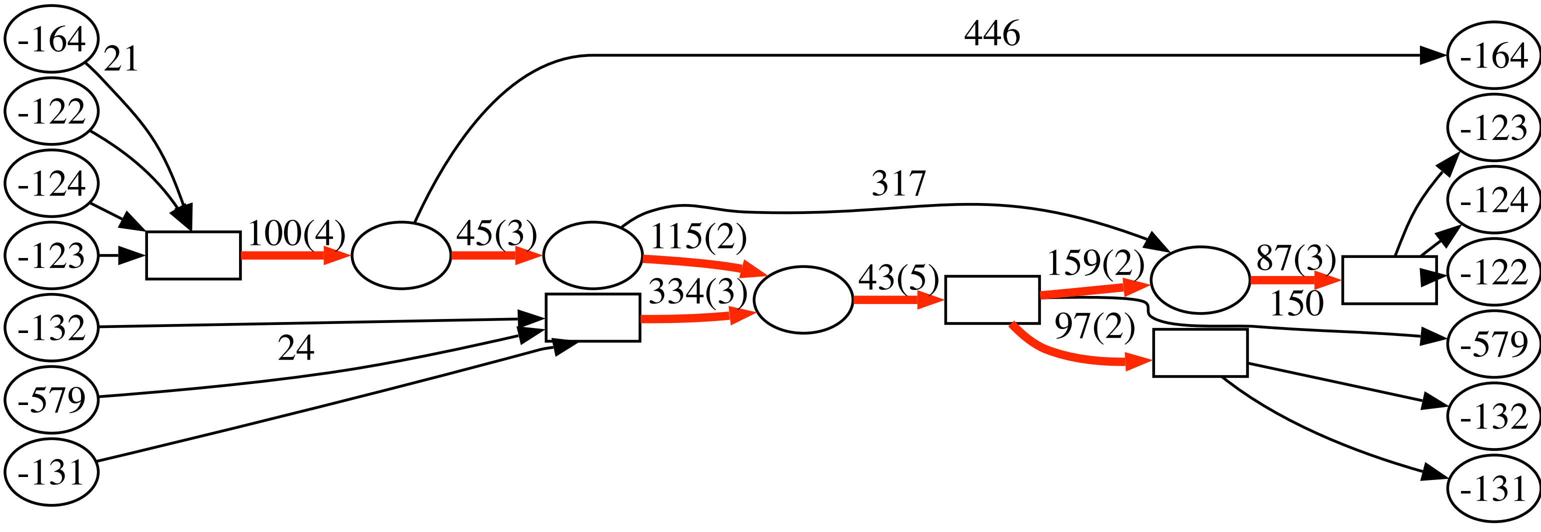

-164
-122
-124
-123
-132
-579
-131
21
24
100(4)
45(3)
446
317
115(2)
334(3)
43(5)
159(2)
97(2)
87(3)
150
-164
-123
-124
-122
-579
-132
-131
